# LTF ameliorates cartilage endplate degeneration by suppressing calcification, senescence and matrix degradation through the JAK2/STAT3 pathway

**DOI:** 10.1111/jcmm.18267

**Published:** 2024-10-11

**Authors:** Tao Li, Yuchi Liu, Jian Cao, Chongzhi Pan, Rui Ding, Jiangminghao Zhao, Jiahao Liu, Dingwen He, Jingyu Jia, Xigao Cheng

**Affiliations:** ^1^ Department of Orthopedics The Second Affiliated Hospital of Nanchang University Nanchang China; ^2^ Institute of Orthopedics of Jiangxi Province The Second Affiliated Hospital of Nanchang University Nanchang China; ^3^ Institute of Minimally Invasive Orthopedics Nanchang University Nanchang China

**Keywords:** calcification, cartilage endplate degeneration, extracellular matrix degradation, JAK2/STAT3 pathway, lactotransferrin, senescence

## Abstract

Intervertebral disc degeneration (IDD)‐induced cervical and lumbar herniations are debilitating diseases. The function of intervertebral disc (IVD) mainly depends on the cartilage endplate (CEP), which provides support and waste removal. Therefore, IDD stems from the degeneration of CEP. Our study shows that the expression of lactotransferrin (LTF), an iron‐binding protein, is significantly decreased in degenerated human and rat CEP tissues. In addition, we found that LTF knockdown promoted calcification, senescence, and extracellular matrix (ECM) degradation in human endplate chondrocytes. Furthermore, the in vivo experiment results confirmed that the JAK2/STAT3 pathway inhibitor AG490 significantly reversed these effects. In addition to investigating the role and mechanism of LTF in CEP degeneration, this study provides a theoretical basis and experimental evidence to improve IDD treatment.

## 1 INTRODUCTION

Intervertebral disc degeneration (IDD) can cause severe neck and lower back pain as well as disability.^1^, ^2^ Among 291 diseases in 187 countries, IDD‐related illness is the leading cause of disability, imposing a serious socioeconomic burden worldwide.^3^, ^4^ The intervertebral disc (IVD) comprises the nucleus pulposus in the center, an annulus fibrosus surrounding it, and cartilage endplates (CEPs) connecting the nucleus pulposus to the vertebral bodies above and below. The IVD is the largest avascular structure in the human body. Cells in the IVD are mainly supplied with nutrients by the CEP transport system, which also plays a role in the maintenance of homeostasis in the IVD microenvironment by eliminating waste. In this regard, CEP thickness is positively correlated with the IDD degree. In addtition, it has been shown that the extracellular matrix (ECM) and cells develop fibrosis and that the thickness of CEP is increased in severely degenerated discs.^5^, ^6^, ^7^, ^8^ In a previous study, ECM degradation in CEPs disrupted water content maintenance, eventually accelerating the degeneration of the entire IVD.^9^ Moreover, research has indicated that CEP degeneration is the IDD‐initiating factor.^10^, ^11^ Therefore, understanding the pathogenesis of CEP degeneration is crucial for developing novel IDD therapies.

Lactotransferrin (LTF) is an iron‐binding protein of the transferrin family. It regulates several roles in various human systems and exerts antibacterial, antioxidant, antitumor, antiviral, immune regulation and anti‐ageing biological effects.^12^, ^13^ Besides regulating lipid metabolism and reducing fat accumulation, LTF promotes osteogenesis and can also reduce oxidative stress (OS) development.^14^, ^15^, ^16^ Furthermore, some scholars explored the role of LTF in IDD, which has been explored largely in relation to its effect on the nucleus pulposus. Findings from studies showed that LTF protects the nucleus pulposus cells.^17^, ^18^ Moreover, LTF can promote chondrocyte proliferation. However, no studies have explored the role of LTF in CEP.

In this study, we analysed LTF expression in normal and degenerated CEP tissues and found that LTF was downregulated in degenerated CEP tissue samples. Subsequently, we explored the role and mechanism of LTF in CEP degeneration. Our findings may offer novel ideas for future IDD treatment using CEPs‐based drugs.

## 2 MATERIALS AND METHODS

### 2.1 Ethics statement

This study was approved by the Ethics Committee of the Second Affiliated Hospital of Nanchang University. Human cervical CEP tissue samples were obtained from patients undergoing discectomy at the Second Affiliated Hospital of Nanchang University. All patients signed written informed consent to allow the use of their tissue specimens for this research.

### 2.2 Clinical specimens

Based on the Pfirrmann grading system that categorizes IVDs according to their appearance on magnetic resonance imaging (MRI), Grade I, II and III were designated as the control group, whereas Grade IV and V were classified as the degenerative group. Human specimens were derived from twenty patients in total. Degenerated human CEP tissues (*n* = 10) were collected from patients undergoing discectomy for degenerative cervical myelopathy (Grade IV–V). For the control group, CEP tissues (*n* = 10) were obtained from patients who underwent surgery for scoliosis, fresh cervical fracture, and spinal cord injury (Grade I–III).^19^ Patients with rheumatoid arthritis (RA), immune diseases, seropositive and negative spondyloarthropathy, thyroid illnesses, tumours and tuberculosis were excluded from the study. The CEP tissues were surgically extracted, rapidly frozen in liquid nitrogen, and stored at −80°C, awaiting further analysis.

### 2.3 Cell culture

The human endplate chondrocyte cell line C28/I2 was cultured in Dulbecco's Modified Eagle Medium (DMEM/F12; Thermo Fisher Scientific, China) with 10% Fetal Bovine Serum (FBS; Thermo Fisher Scientific) and 1% penicillin–streptomycin (Invitrogen) at 37°C and 5% CO_2_ in humid conditions.

### 2.4 Haematoxylin and eosin staining and Safranin‐O staining

Human CEPs were fixed in 10% formalin for 2 days and decalcified in 10% EDTA for 45 days. The fixed samples were subsequently embedded in paraffin and then sectioned into 6 μm/shee sizes for haematoxylin and eosin and Safranin O staining.

### 2.5 Immunofluorescence (IF) staining

The endplate chondrocytes were seeded on grass coverslips, fixed with 4% paraformaldehyde in phosphate‐buffered saline (PBS) for 20 min, and then washed with PBS. Following that, the coverslips were treated with 1% Triton X‐100 in PBS for 10 min and then washed. Next, the endplate chondrocytes were incubated overnight with anti‐P21, anti‐Runx2, and anti‐MMP13 antibodies at 4°C and then washed four times with PBS. Subsequently, the cells were incubated with secondary antibodies for 2 h and then washed with PBS. They were then stained with DAPI for 10 min and mounted with a slide mounting medium for future microscopic examination. All antibodies were diluted with 5% bovine serum albumin (Sangon Biotech, Shanghai, China). The slides were observed under a confocal microscope (Leica Microsystems, Wetzlar, Germany), and pictures were analysed using Image J software.

### 2.6 Immunohistochemistry (IHC) staining

Immunohistochemistry staining was performed to measure MMP13, Runx2, and P21 expression. The slices were first dewaxed in xylene, dehydrated in graded ethanol and incubated in 3% H_2_O_2_ at 37°C for 10 min. This was followed by washing in PBS for 5 min three times and boiling in a 0.01 M citric acid buffer for antigen retrieval (95°C, 15–20 min). It was blocked in goat serum for 10 min at 37°C. Next, the slices were incubated with the following primary antibodies: anti‐MMP13 (1:50), anti‐Runx2 (1:50), and anti‐P21 (1:200) all purchased from Proteintech (WuHan, China) at 4°C overnight and then with a biotin‐labelled secondary antibody (Proteintech) for 30 min at 37°C. Finally, the slices were counterstained with haematoxylin and observed under a light microscope.

### 2.7 Western blotting assessment

The total protein was obtained using a protein extraction kit (APPLYGEN, Beijing, China) following the standard protocol. About 20 μg of proteins were loaded in each lane and separated by 10% Sodium‐Dodecyl Sulfate Poly‐Acrylamide Gel Electrophoresis (SDS‐PAGE) before transferring to Polyvinylidene Fluoride (PVDF) membranes. The proteins were subsequently blocked with 5% non‐fat milk for 2 h and then incubated overnight with the primary antibodies at 4°C, followed by incubation with the corresponding secondary antibodies for 2 h at room temperature (RT). The primary antibodies (1:1000) used included LTF (10933‐1‐AP), P16 (910883‐1‐AP), P21 (10355‐1‐AP), P53 (10442‐1‐AP), Runx2 (20700‐1‐AP), and OPN (22952‐1‐AP) (Proteintech), as well as Aggrecan, Collagen II, MMP13, JAK2 (ab108596), p‐JAK2 (ab32101), STAT3 (ab68153), p‐STAT3 (ab267373) (Abcam, United States) and goat anti rabbit‐IgG (1:5000) (15,015; Proteintech). The membranes were then rinsed with Tris‐Buffered Saline and Tween 20 three times and visualized using the electrochemiluminescence plus reagent (Invitrogen). The intensity of the blots was then quantified with the Image Lab 3.0 software (BioRad, Shanghai, China).

### 2.8 Si‐RNA transfection

The LTF siRNA was purchased from RiboBio (Guangzhou, China). Cells were cultured in six‐well plates for 24 h to achieve a 60%–70% density. Subsequently, 50 nM of a negative control or LTF siRNA was added using the riboFECT™ CP Transfection reagent per the manufacturer's instructions. After 48 h, cellular lysates were obtained through centrifugation to analyse the expression of genes of interest.

### 2.9 LTF Adenovirus and Infection

The adenoviruses expressing LTF were purchased from Genechem (Shanghai, China). Cells were cultured in DMEM with 10% FBS at 37°C and 5% CO_2_ to achieve a density of 60%–70%. Next, cells were infected with the Ad‐LTF virus for 12 h and incubated as previously described for 36 h.

### 2.10 Von Kossa staining

Calcification was assessed using the Von Kossa staining kit (Beyotime, Shanghai, China). Cells were washed two times with PBS and fixed in 95% ethanol at RT for 10 min before washing with PBS. The cells were incubated in a Von Kossa silver solution for 1 min and washed two times with PBS. After incubation under UV light for 10 min and washing with distilled water, the endplate chondrocytes were mixed with 1 mL Hypo solution for 1 min. Next, they were counterstained with 1 mL haematoxylin solution for 2 min and counterstained with 1 mL eosin solution for 1 min. Images were then captured under a light microscope after washing and drying. The calcium nodules exhibited a dark black appearance.

### 2.11 SA‐β‐gal staining

Cell senescence was assessed with a senescence‐associated β‐galactosidase (SA‐β‐gal) staining kit (Beyotime). The entire experiment was performed following the manufacturer's instructions. The senescent endplate chondrocytes were stained blue to show increased SA‐β‐gal activity.

### 2.12 Construction and treatment of rat IDD model

The rat IDD model was constructed through the needle puncture method previously described by Luo et al.^20^ using forty (eight‐week‐old) adult Sprague–Dawley (SD) rats acquired from Changsha Tianqin Biotechnology. Briefly, general anaesthesia was induced using pentobarbital 1% sodium (100 mg/kg). Under fluoroscopy guidance, a 31 G needle was used to puncture the tail Co8/9 from the dorsal side, passing through the annulus fibrosus into the NP region for about 1.5 mm, then rotated 180° in the axial direction and held for 30 s. The animals were randomly divided into four groups (*n* = 10): Control (received no treatment), Degeneration (underwent tail acupuncture and was injected with 2 μL saline), Degeneration+AG490 [underwent tail acupuncture and was injected with 2 μL AG490 (MedChemExpress, Shanghai, China)], and Degeneration+ LTF [underwent tail puncture and was injected with 2 μL rehLTF (32 μM) (Sino Biological, Beijing China)].

### 2.13 Radiological examination

We also captured X‐ray images and determined the disc height and Disc Height Index (DHI). Changes in DHI (calculated as DHI % = post‐DHI/pre‐DHI × 100%) were used to evaluate disc degeneration. Post‐DHI and pre‐DHI were the post‐operation and pre‐operation DHI, respectively.

### 2.14 Statistical analysis

All statistical analyses were performed using SPSS 23.0 software (IBM, USA). All experiments were repeated at least three times. Intergroup differences were compared using one‐way analysis of variance (ANOVA) and the Student's *t*‐test. Results were presented as mean (M) ± standard deviation (SD), and those with *p* < 0.05 were considered statistically significant.

## 3 RESULTS

### 3.1 The expression of LTF is decreased in human degenerated CEP

**FIGURE 1:**
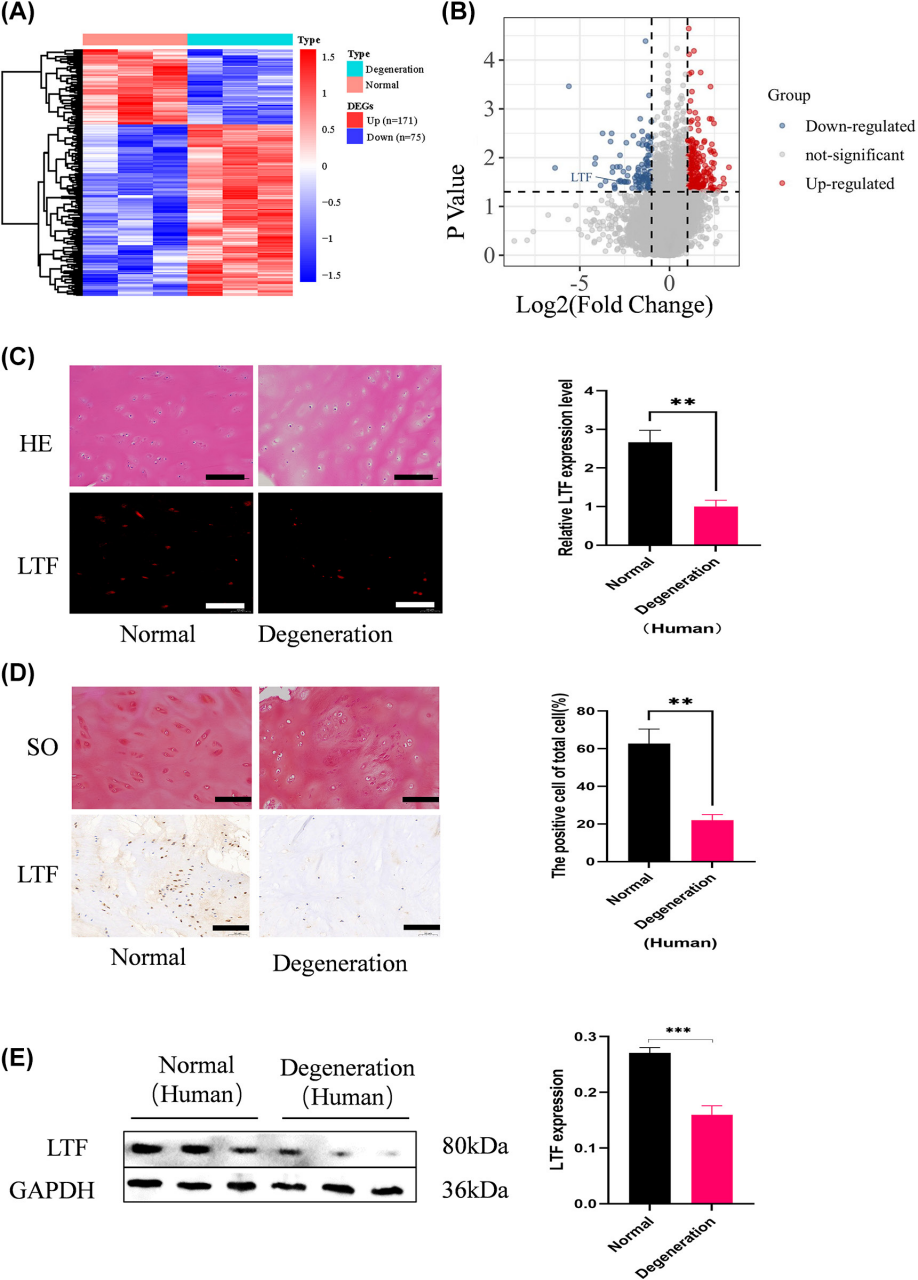
LTF expression decreased in human degenerated CEPs. (A) Heat map of differentially expressed mRNA in the two groups (fold change >2; *p* < 0.05, 171 vs. 75). (B) Volcano plot showing differentially expressed mRNA with red and blue indicating downregulated and upregulated mRNA, respectively. (C) Representative haematoxylin and eosin staining, IF staining, and LTF quantification in the two human CEP tissue groups. (D) Safranin O staining, IHC staining, and quantification (Scale Bar =100 μm0. (E) WB analysis of LTF protein expression and quantification. All data are expressed as mean ± SD (*n* = 3); **p < 0.01, ***p < 0.001.

According to our previous study,^21^ which we can be found in the gene expression omnibus (GEO) database, accession number GSE153761, we mapped out the mRNA expression profile in normal and degenerated CEP samples through hierarchical clustering (Figure 1A) and illustrated the mRNA expression differences using a volcano plot (Figure 1B). Compared to normal tissue samples, 246 mRNAs were found to be differentially regulated. Among these, 171 mRNAs were upregulated and 75 were downregulated in the degenerative group. Through reviewing existed literatures, we found that LTF plays a role in IDD, it as well can regulate the function of chondrocytes. Therefore, from the downregulated mRNAs, we selected LTF for further investigation.^17^, ^18^, ^22^, ^23^

Normal and degenerative human CEP tissues were collected as before described to investigate the role of LTF in cervical CEP degeneration. The tissue groups were compared using immunofluorescence (IF) and immunohistochemistry (IHC) staining (Figure 1C,D). Results showed that LTF expression was lower in the degenerate CEP samples compared with normal. Next, we performed western blot (WB) experiment using tissue extracts from the normal and degenerated specimens. It was observed that LTF was downregulated in isolated human cartilage tissues from the degenerated group (Figure 1E). These findings imply that LTF levels were downregulated in the degenerative cervical CEPs.

### 3.2 LTF downregulation induces calcification, senescence, and ECM degradation in endplate chondrocytes

**FIGURE 2:**
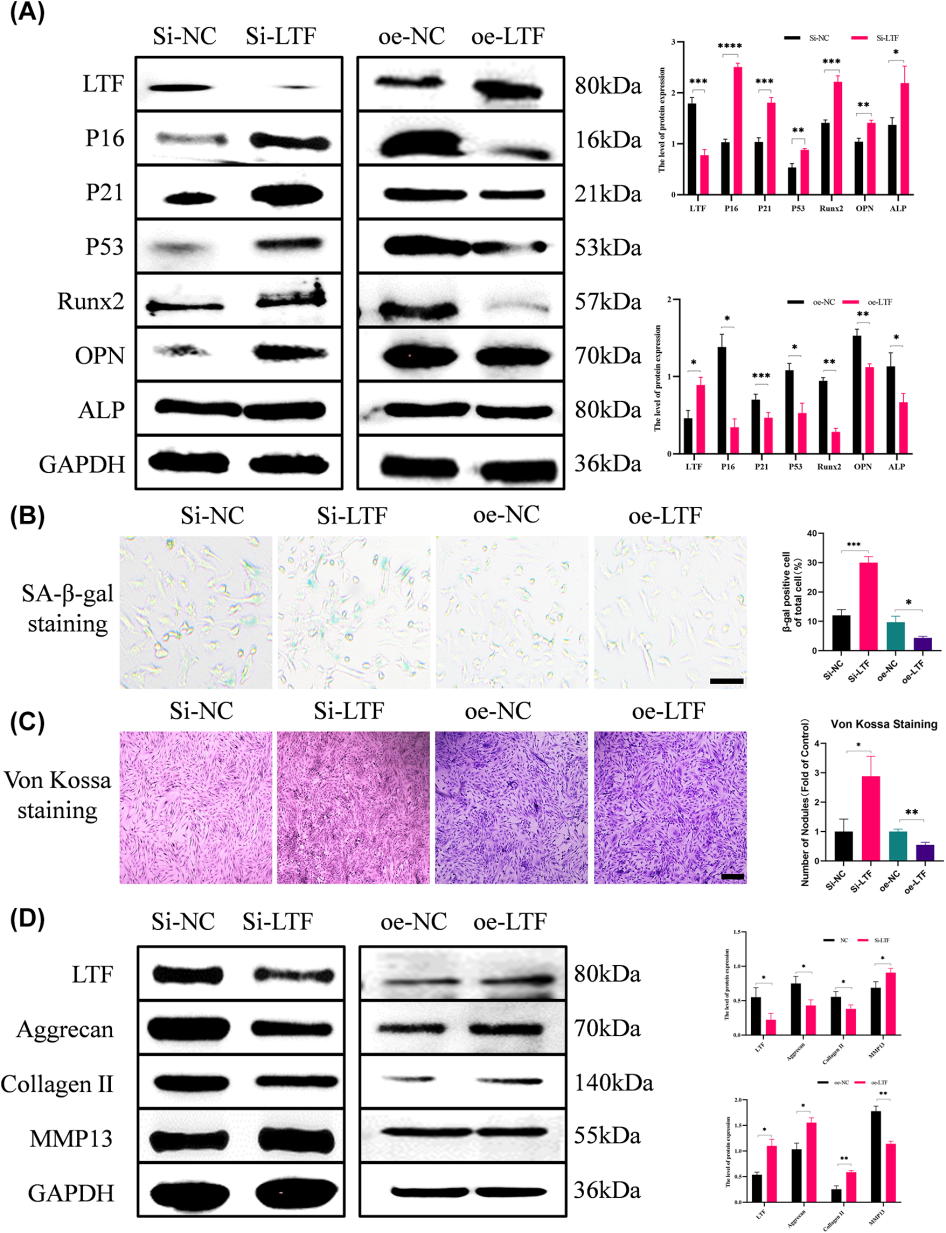
Function of LTF downregulation in endplate chondrocytes. (A) WB analysis and quantification revealed the LTF levels, senescence markers (P16, P21, and P53), and classification (Runx2 and OPN) after si‐LTF and oe‐LTF treatment. (B) SA‐β‐gal staining and quantification of positive cells as a proportion of total cells in these four groups (Scale bar = 100 μm). (C) Von Kossa staining and quantification of nodules in these four groups (Scale bar = 100 μm). (D) WB analysis of LTF, Aggrecan, Collagen II, and MMP13 in the presence of si‐NC, si‐LTF. oe‐NC and oe‐LTF. All data are presented as mean ± SD (*n* = 3); *p < 0.05, **p < 0.01, ***p < 0.001, ****p < 0.0001.

To explore the effect of LTF on endplate chondrocytes, we downregulate and upregulate the expression of LTF in chondrocyte cell lines. Calcification in CEPs hinders IVD metabolism, closely linked to IVD degeneration. To investigate this, we examined calcification and the expression of calcification‐related proteins (Runx2, OPN and ALP) through Von Kossa staining and WB, respectively. Our results showed a negative correlation between the expression level of LTF and that of calcification‐related makers (Figure 2A). Von Kossa staining revealed that si‐LTF promoted calcium nodule formation in endplate chondrocytes, whereas calcium nodule formation decreased in the oe‐LTF group (Figure 2C).

Furthermore, CEP degeneration correlates with endplate chondrocyte senescence and ECM degradation. Therefore, we investigated the impact of LTF knockdown and overexpression on senescence and ECM degradation. The levels of P16, P21 and P53 were used as indicators of endplate chondrocyte senescence, which were found to be upregulated in si‐LTF‐treated endplate chondrocytes and be downregulated in oe‐LTF‐treated. (Figure 2A). These findings were confirmed by SA‐β‐gal staining (Figure 2B). Additionally, we explored Collagen II, Aggrecan and MMP13 levels to assess the effects of LTF on ECM degradation. The results indicated that LTF knockdown downregulated ECM generation‐associated proteins (Collagen II and Aggrecan) and upregulated ECM degradation‐associated protein MMP13, with opposite results observed with oe‐LTF treatment (Figure 2D). Overall, LTF knockdown induces calcification, senescence, and ECM degradation in endplate chondrocytes.

### 3.3 LTF regulates CEP degeneration via the JAK2/STAT3 signalling pathway

**FIGURE 3:**
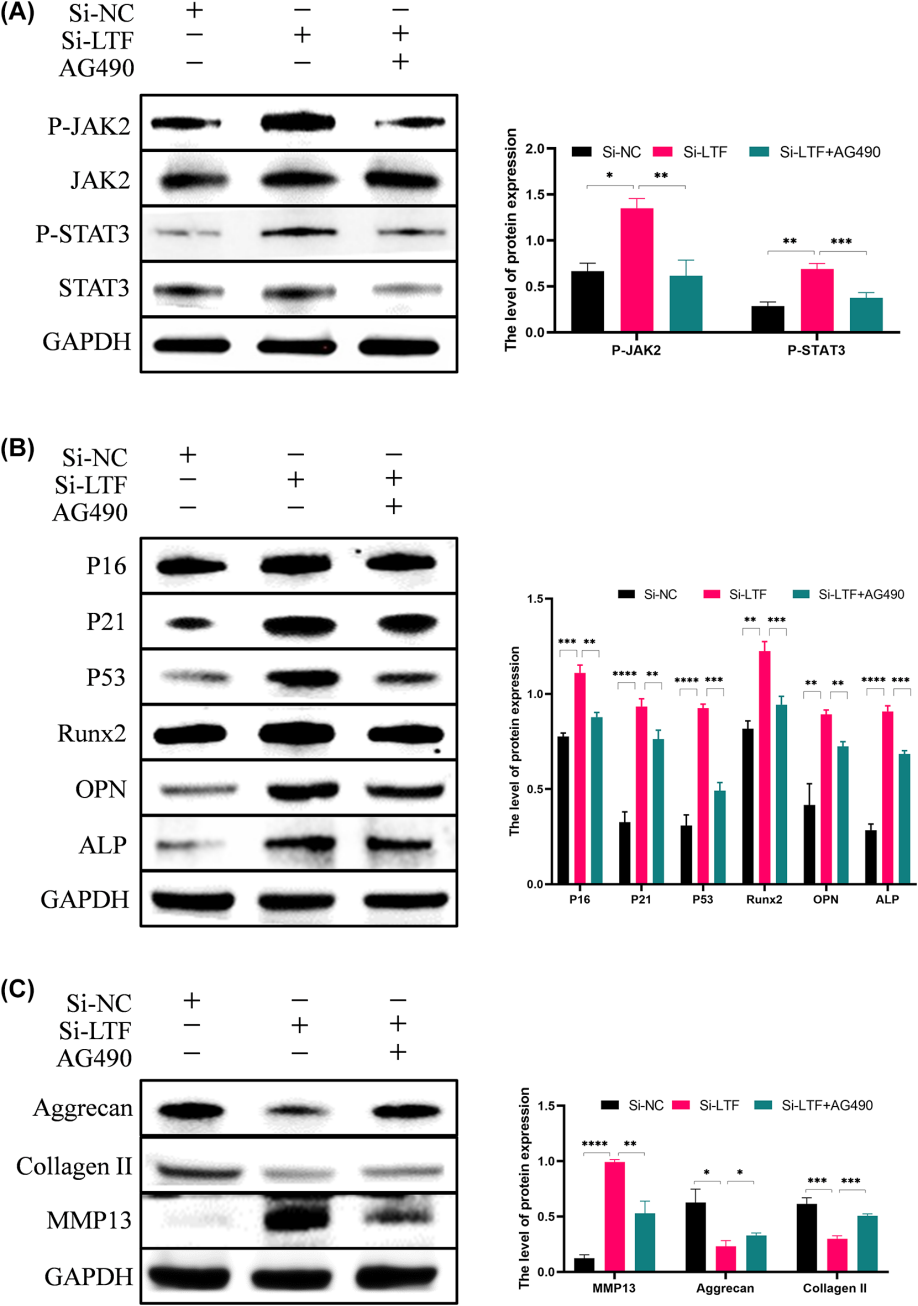
LTF regulates endplate chondrocyte function via the JAK2/STAT3 pathway. (A) WB analysis and quantification of p‐JAK2 and p‐STAT3 levels after si‐NC, si‐LTF and si‐LTF + AG490 treatments. (B) WB analysis and quantification of Runx2, OPN, ALP, P16, P21, and P53 levels in the three groups. (C) WB analysis of MMP13, Collagen II, and Aggrecan levels in different groups. All data are shown as mean ± SD (*n* = 3); *p < 0.05, **p < 0.01, ***p < 0.001, ****p < 0.0001.

**FIGURE 4:**
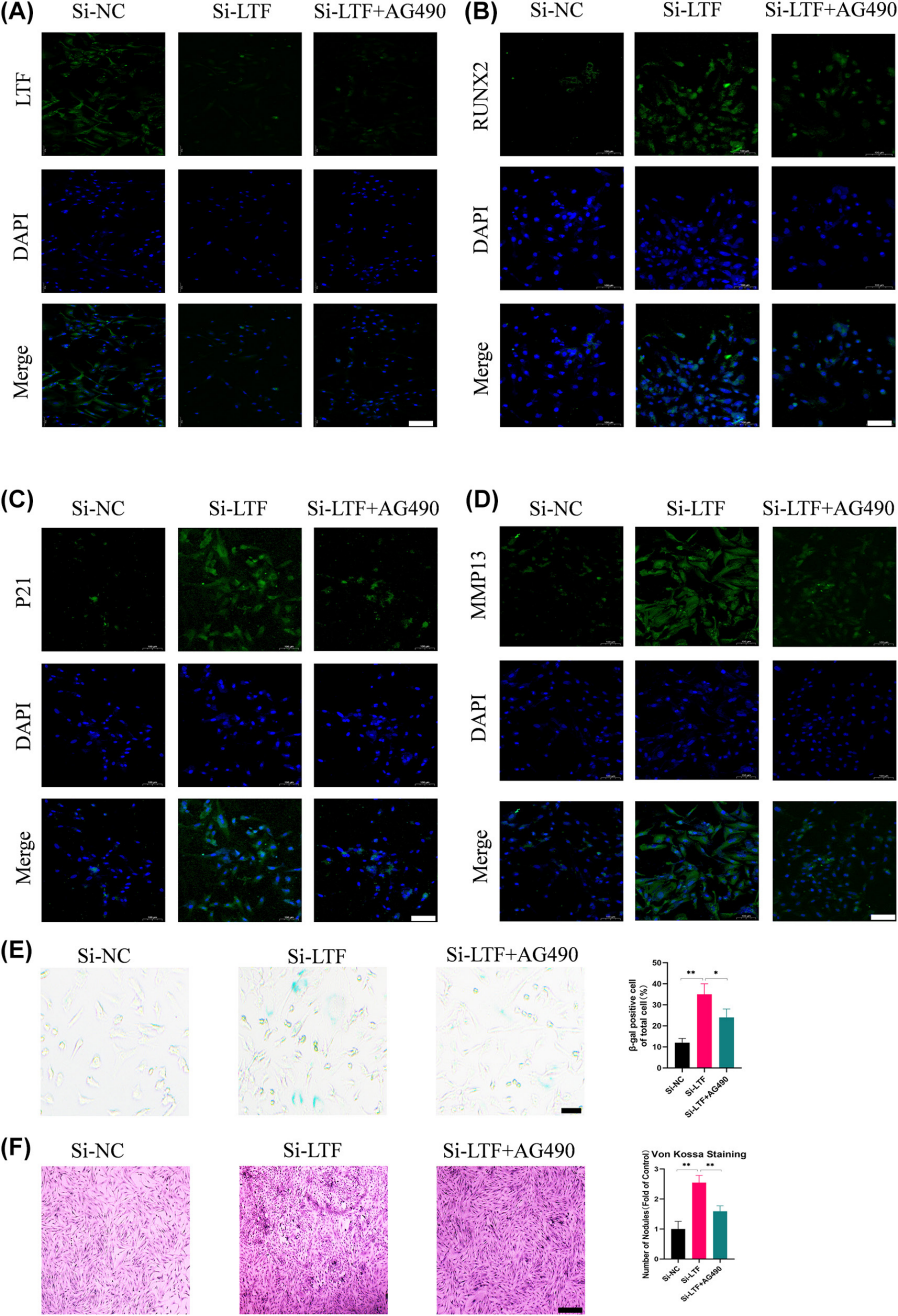
LTF induced functional alterations in endplate chondrocytes, suppressed the JAK2/STAT3 pathway, and reversed the impact of si‐LTF treatment. (A–D) IF staining of LTF, Runx2, P21, and MMP13 in a human chondrocyte (Scale bar = 100 μm). (E) SA‐β‐gal staining and quantification of positive cells as a proportion of total cells in the three groups (Scale bar = 100 μm). (F) Von Kossa staining and quantification of nodules in the three groups (Scale bar = 100 μm) (Scale bar = 100 μm). All data are presented as mean ± SD (*n* = 3); *p < 0.05, **p < 0.01.

Previous studies have explored the pathways that may contribute to endplate chondrocyte calcification, such as MAPK/NFκB pathway or Nrf‐2/HO‐1 pathway and so on.^24^, ^25^ In this study, we select the Janus kinase 2/signal transducer and the activator of transcription 3 (JAK2/STAT3) signalling pathway to explore. The JAK2/STAT3 pathway has been reported to participate in functional alterations in chondrocytes.^26^ Therefore, we investigated whether LTF regulates endplate chondrocyte functions via the JAK2/STAT3 signalling pathway. The results demonstrated an increase in the phosphorylation of JAK2 and STAT3 protein levels in the si‐LTF group (Figure 3A). For further analysis, the JAK2/STAT3 pathway inhibitor AG490 was introduced, and p‐JAK2 and p‐STAT3 were downregulated in endplate chondrocytes. The inhibitor AG490 targeting the JAK2/STAT3 pathway significantly reversed the si‐LTF‐induced effects, including the downregulation of collagen II and Aggrecan and the upregulation of Runx2, OPN, ALP, P16, P21, P53 and MMP13 (Figure 3B,C). This observation was further supported by immunofluorescence analysis of Runx2, P21 and MMP13 (Figure 4A–D). Additonally, SA‐β‐gal staining and Von Kossa staining results were also reversed by AG490 (Figure 4E,F). Overall, si‐LTF induces calcification, senescence, and ECM degradation in endplate chondrocytes via the JAK2/STAT3 signalling pathway.

### 3.4 LTF alleviates IDD in rats

**FIGURE 5:**
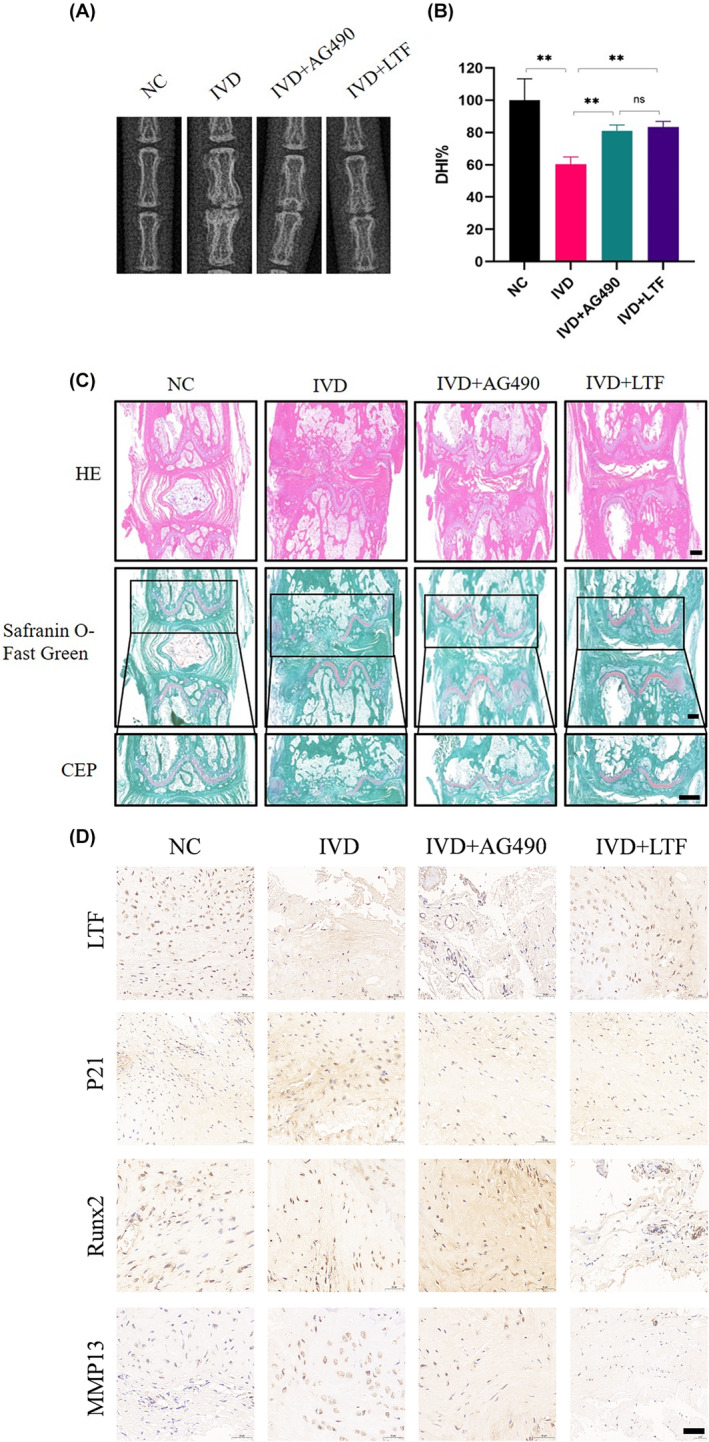
LTF supplementation to the IVD alleviates IDD in rats. (A, B) Digital X‐ray images of rat tails and DHI % from different experimental groups. (C) Representative HE staining and Safranin O‐fast green staining images after four different treatments (Scale bar = 500 μm). (D) IHC staining of CEPs from rat tails in the four groups (Scale bar = 500 μm). All data are expressed as mean ± SD (*n* = 3); **p < 0.01.

The mechanism of LTF was evaluated in vivo using a surgically IVDD model established by needle puncture in rats. AG490 was employed to target the JAK2/STAT3 pathway. Histological analysis revealed that compared to the control group, blockade of the JAK2/STAT3 pathway partially restored the degeneration of the intervertebral discs induced by puncture. Moreover, injecting recombinant human Lactotransferrin (rehLTF) also alleviated disc degeneration caused by the acupuncture model. Compared to the IVD group, the IVD + AG490 and IVD + rehLTF groups had less calcification on the cartilage surface and lower joint space stenosis (Figure 5A–C). Immunohistochemical staining of rat tail CEPs revealed that rehLTF downregulated the expression of p21, Runx2, and MMP13 (Figure 5D), implying that LTF lowered calcification, senescence, and ECM degradation in endplate chondrocytes. Furthermore, AG490 can reverse CEP degeneration, suggesting that LTF ameliorates IVD in rats through the JAK2/STAT3 pathway. Moreover, immunofluorescence staining showed similar results (Figure S1A).

## 4 DISCUSSION

With a lifetime cervical‐related pain incidence greater than 65%, disc degeneration is the leading cause of cervical, dorsal, and radicular pain.^27^, ^28^ The cartilage endplate, a crucial component of the intervertebral disc, has been a subject of intense research in the context of intervertebral disc degeneration.^29^ Studies by Volkan Emre Arpinar et al. revealed a positive correlation between high magnetic resonance signals in the cartilage endplate and the severity of disc degeneration. Additionally, research by Gullbrand SE et al. demonstrated that abnormal remodelling of the cartilage endplate facilitates the transport of small molecules into the intervertebral disc, representing a significant factor in disc degeneration.^5^ Calcification in CEPs is a key factor in IDD onset and development. According to research, IDD is often accompanied by CEP thickening, increasing endplate cell senescence, and enhancing ECM fibrosis.^30^, ^31^ Notably, LTF participates in bone formation, anti‐ageing processes, and ECM degradation.^32^, ^33^ In this line, our experimental results imply that LTF can alleviate endplate chondrocyte calcification, senescence, and ECM degradation, thereby improving IDD.

A previous study reported that LTF exerts excellent osteogenic effects in osteoblasts and it is a bioactive molecule that can be used for bone regeneration.^14^ To date, the association between LTF and calcification remains unexplored in the existing literature. In this study, we observed that LTF knockdown resulted in the upregulation of calcification‐related markers in endplate chondrocytes, as evidenced by increased levels of Runx2 and OPN, a finding further supported by Von Kossa staining. (Figure 2A–C). The LTF anti‐ageing effect has been demonstrated in various diseases, and Park^34^ confirmed that LTF can protect the ageing of human mesenchymal stem cells (MSCs) and significantly improve their therapeutic effect. Additionally, Chen et al. discovered that LTF can promote osteogenesis and inhibit bone resorption by lowering osteoblast senescence.^35^ Consistent with previous research, this study reflected the LTF anti‐ageing effect in CEPs. Endplate chondrocytes with low LTF expression exhibited higher senescence‐related protein (P16, p21, p53) levels and proportion of positive cells in the β‐galactose senescence‐related staining. (Figure 2A–D). Ellman et al. discovered that LTF could promote the anabolism of nucleus pulposus cells through BMP7 during disc degeneration.^17^ Furthermore, Kim et al. confirmed the LTF anticatabolic properties in the IVD.^18^ Adding to the existing literature, this study explored the LTF anti‐matrix degradation effect in CEPs.

The JAK2/STAT3 signalling pathway is critically involved in regulating the formation of various tumours and cerebral ischemia and Alzheimer's Disease (AD) treatment.^36^, ^37^, ^38^ However, the involvement of the JAK2/STAT3 signalling pathway in IDD is rarely explored. Zhang et al. demonstrated that 17‐AAG could alleviate IDD via attenuating inflammation and the ECM degradation of nucleus pulposus cells by inhibiting the JAK2/STAT3 pathway.^39^ We found that LTF knockdown reduced the suppression of the JAK2/STAT3 signalling pathway, increasing calcification, senescence, and ECM degradation in endplate chondrocytes. However, the JAK2/STAT3 signalling inhibitor AG490 reversed these effects (Figures 3 and 4). To investigate the role of LTF in regulating CEP degeneration and its potential therapeutic effect on IDD, we utilized an in vivo rat model of IDD induced by caudal vertebrae acupuncture, while concurrently blocking the JAK2/STAT3 signalling pathway. We discovered that the exogenous supplementation of rehLTF can suppress calcification, senescence, and ECM degradation in CEPs via the JAK2/STAT3 signalling pathway, thereby alleviating CEP degeneration and delaying IDD progress.

Additionally, the above experiments revealed that LTF can ameliorate CEP degeneration and IDD. It is noteworthy that this study had several limitations. First, the effects and mechanisms of LTF overexpression in endplate chondrocytes were not investigated. Second, we did not elucidate whether LTF regulates JAK2 and STAT3 expression directly or indirectly. Third, we used the SD rat model, which may not fully represent the characteristics of human IDD. These issues warrant additional research.

## 5 CONCLUSION

Our study employed ceRNA microarray technology to compare mRNA expression levels in two groups of tissue samples, revealing decreased LTF expression in degenerated CEPs. Subsequent downregulation of LTF led to increased phosphorylation of JAK2 and STAT3, culminating in enhanced CEP calcification, senescence and ECM degradation, thereby contributing to disc degeneration. These findings provide new insights and a theoretical foundation for more effective treatments targeting IDD.

## AUTHOR CONTRIBUTIONS

**Xigao Cheng:** Conceptualization (supporting); funding acquisition (lead); supervision (equal); visualization (equal). **Tao Li:** Conceptualization (lead); investigation (equal); writing – original draft (equal). **Yuchi Liu:** Investigation (equal); writing – original draft (equal); writing – review and editing (lead). **Jian Cao:** Investigation (equal). **Chongzhi Pan:** Writing – review and editing (equal). **Rui Ding:** Data curation (equal). **Jiangminghao Zhao:** Investigation (equal). **Jiahao Liu:** Data curation (equal). **Dingwen He:** Project administration (equal); supervision (equal). **Jingyu Jia:** Methodology (supporting); project administration (equal); supervision (equal).

## FUNDING INFORMATION

National Natural Science Foundation of China, Grant/Award Number: 82060403; Thousand Talents Program of Jiangxi Province, Grant/Award Number: JXSQ2019201026; Interdisciplinary Innovation Fund of Natural Science, NanChang University.

## CONFLICT OF INTEREST STATEMENT

The authors declare no conflict of interest.

## Supporting information


Figure S1.

## Data Availability

The data that support the findings of this study are available from the corresponding author upon reasonable request. The datasets presented in this study can be found in online repositories. The name of the repository and accession number can be found in the article.
