# Supplementary figures and images for: LTF ameliorates cartilage endplate degeneration by suppressing calcification, senescence and matrix degradation through the JAK2/STAT3 pathway
Source: J Cell Mol Med. 2024 Oct 11;28(19):e18267. doi: 10.1111/jcmm.18267 (PMC11467740; doi:10.1111/jcmm.18267)

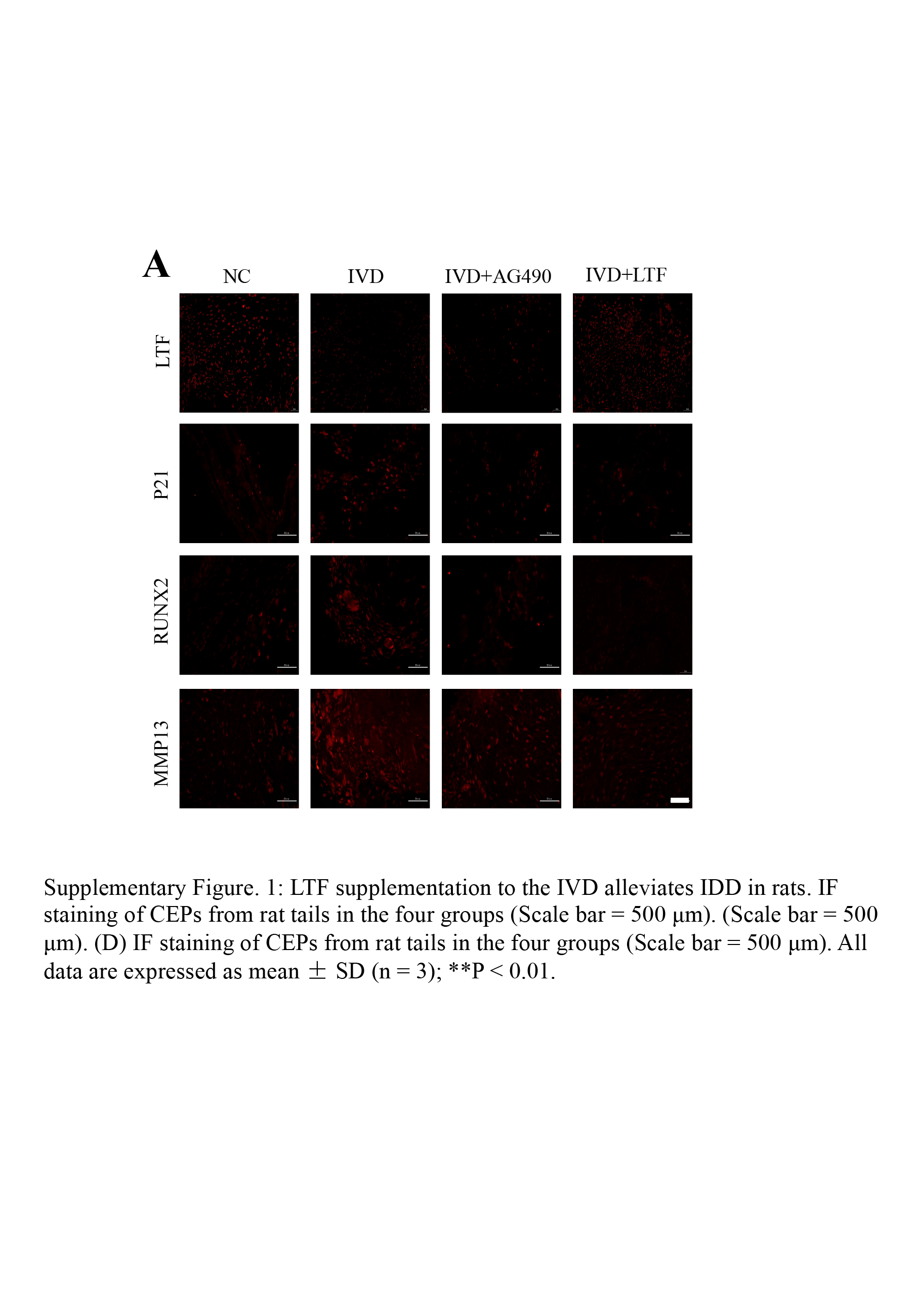

Supplement: Supplementary file 1 — Figure S1. [file JCMM-28-e18267-s001.tif]
